# Nanoporous composites prepared by a combination of SBA-15 with Mg–Al mixed oxides. Water vapor sorption properties

**DOI:** 10.3762/bjnano.5.136

**Published:** 2014-08-07

**Authors:** Amaury Pérez-Verdejo, Alvaro Sampieri, Heriberto Pfeiffer, Mayra Ruiz-Reyes, Juana-Deisy Santamaría, Geolar Fetter

**Affiliations:** 1 Benemérita Universidad Autónoma de Puebla, Facultad de Ingeniería Química, Ciudad Universitaria, 72570, Puebla, PUE, Mexico; 2 Instituto de Investigaciones en Materiales, Universidad Nacional Autónoma de México, Circuito exterior s/n, Cd. Universitaria, Del. Coyoacán, 04510, México DF, Mexico; 3 Benemérita Universidad Autónoma de Puebla, Facultad de Ciencias Químicas, Ciudad Universitaria, 72570, Puebla, PUE, Mexico

**Keywords:** calcined Mg–Al hydrotalcite, nanoporous composites, SBA-15, vapor sorption

## Abstract

This work presents two easy ways for preparing nanostructured mesoporous composites by interconnecting and combining SBA-15 with mixed oxides derived from a calcined Mg–Al hydrotalcite. Two different Mg–Al hydrotalcite addition procedures were implemented, either after or during the SBA-15 synthesis (in situ method). The first procedure, i.e., the post-synthesis method, produces a composite material with Mg–Al mixed oxides homogeneously dispersed on the SBA-15 nanoporous surface. The resulting composites present textural properties similar to the SBA-15. On the other hand, with the second procedure (in situ method), Mg and Al mixed oxides occur on the porous composite, which displays a cauliflower morphology. This is an important microporosity contribution and micro and mesoporous surfaces coexist in almost the same proportion. Furthermore, the nanostructured mesoporous composites present an extraordinary water vapor sorption capacity. Such composites might be utilized as as acid-base catalysts, adsorbents, sensors or storage nanomaterials.

## Introduction

Multifunctional nanomaterials are designed to satisfy specific ranged sets of performance requirements. The particular suitability of these materials as target materials depends on their composition, micro or nanostructure, porosity, acid-base character or biocompatibility [1]. The synthesis of nanocomposites can be achieved in a variety of different ways, such as nanocasting or self-assembling templates [2–3]. However, the design of nanocomposite materials is usually a challenging task, as it is often necessary to employ several steps with complex preparation methods. For instance, composite materials can be prepared by a combination of inorganic with organic moieties or hybrid materials, to obtain a hydrophobic–hydrophilic character, among other physicochemical properties. The most studied multifunctional materials are the hybrids, which are good candidates for biomedical applications, e.g., biosensors, artificial bonds and bioadsorbents [4–5]. Instead, a few works report the design of purely inorganic composite materials. For example, basic and acidic materials such as hydrotalcite and hydroxyapatite can be combined to produce composite materials whose structure, texture and morphology are unique and determined by the interaction between them [6–7]. More specifically, these interactions determine the porosity or the surface area and the particle size. SBA-15 and MCM-41 have also been successfully used as hosts to incorporate hydroxyapatite nanocrystals to obtain active composites acting as efficient fluoride adsorbents from contaminated water [8]. Furthermore, the in situ SBA-15, previously modified with Si–CH_3_ and then functionalized with Mg and Al nitrate salts, has promoted the nanocrystal growth of Mg–Al hydrotalcite on the pore walls of the SBA-15 [9]. This composite presented a high catalytic activity in the acetone condensation at 273 K. Moreover, a basic composite has been prepared from SBA-15 with MgO and tested as a drug delivery controller material or as a basic catalyst [10]. Recently, Habib et al. [11] synthesized ZSM-5/SBA-15 composites by a microwave-assisted zeolitisation. These composites presented highly specific surface areas and a pore volume with narrow porous size distributions. Still, it is necessary to improve the preparation methods to obtain micro and mesoporous nanostructured composites more easily, with short preparation times, lower overall costs, better textural properties, and highly dispersed active metals. Anionic clays, or simply hydrotalcites, are good candidates to be combined homogenously with mesoporous siliceous materials to improve their physicochemical properties. Hydrotalcites are lamellar materials with basic properties, but with a relative low surface area and poor mesoporosity [12]. Thus, large molecules accessibility toward active sites is a challenging goal. Instead, siliceous ordered mesoporous materials present high surface areas and narrow pores size distributions in the mesoporosity range, but with a poor surface reactivity [13]. If hydrotalcites can be conveniently combined with SBA-15, a novel kind of nanoestructured porous composites may be obtained, and the hydrophilic character of the SBA-15 could be improved with the Mg–Al species. Indeed, the water affinity of these materials has gained importance in certain adsorption processes, for example the process reported by Zhou et al. [14], which the methane amount storage SBA-15 increases with water content. This work proposes two straightforward ways to prepare nanostructured porous composites by interconnecting and combining Mg–Al hydrotalcite with SBA-15. Furthermore, the adsorption–desorption behavior of water vapor is presented to evaluate their hydrophilic character and sorption capacity.

## Results and Discussion

### Textural properties

**Figure 1:**
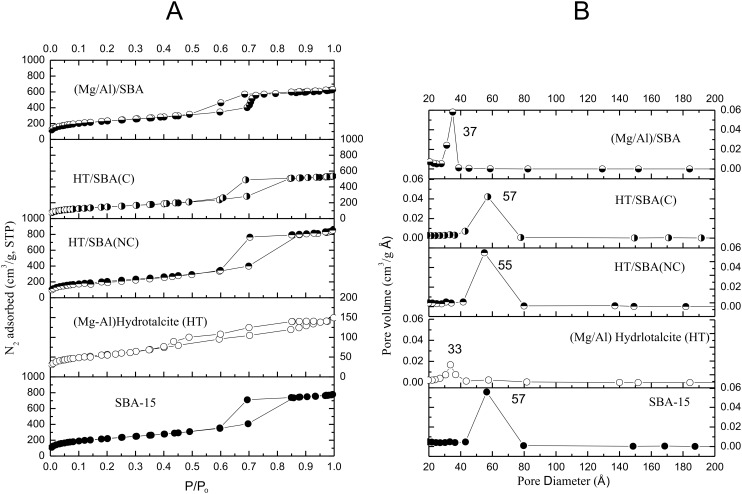
N_2_ adsorption–desorption isotherms (A) and pore diameter distribution (B) of the composites, SBA-15 and HT.

Table 1 summarizes the main textural results obtained by X-ray diffraction (Supporting Information File 1) and N_2_ adsorption–desorption isotherms (Figure 1A).

**Table 1:** Textural properties and Mg/Al molar ratio of the calcined samples.^a^

		Precursors		Composites		
		
		SBA-15	calcined Mg–Al hydrotalcite (HT)	post-synthesis HT/SBA(NC)	post-synthesis HT/SBA(C)	in situ (Mg/Al)/SBA

*S*_BET_	m^2^/g	785	200	734	523	806
*S*_mic_		74	21	37	31	316
*S*_mes_		640	102	606	441	481
*S*_ext_		71	77	91	50	9
*V*_Mic_	cm^3^/g	0.03	0.01	0.01	0.01	0.15
*V*_mes_		1.02	0.19	1.11	0.71	0.36
Ø_p_	nm	5.7	3.3	5.5	5.7	3.7
*d*_100_		9.5	—	10.8	10.3	7.4
AWT		5.3	—	7.0	6.2	4.8
Mg/Al	molar ratio	—	2.03	1.85	1.95	1.40

^a^Ø_p_ = average mesoporous diameter; *d*_100_ = interlaminar distance determined by X-ray diffraction; AWT = average wall-thickness [(2*d*_100_/√3 – Ø]; *S*_mic_ = microporous surface; *S*_mes_ = mesoporous surface; *S*_ext_ = external surface, *V*_mic_,*V*_mes_ = micro and mesopore volumes; *S*_BET_ = *S*_mic_ + *S*_mes_ + *S*_ext_. Mg/Al molar ratio was determined from atomic absorption spectroscopy analysis.

All isotherms are Type IV according to IUPAC classification. Both post-synthesis prepared composites and the SBA-15 show H1 hysteresis loops due to the N_2_ capillary condensation. It also denotes that the pores present a cylindrical form and narrow size distribution (Figure 1B).

Calcined Mg–Al hydrotalcite shows a N_2_ adsorption–desorption isotherm with a wide hysteresis loop (H3 type) related to pores formed by lamellar structures. However, the in situ sample (Mg/Al)/SBA shows an isotherm with H2 type hysteresis that indicates an interconnection of mesoporous by microporous. The 734 m^2^/g BET surface area of the post-synthesis composite, HT/SBA(NC), is higher than that of the calcined Mg–Al hydrotalcite (200 m^2^/g) and quite similar to the BET surface area of the SBA-15 (785 m^2^/g). In this composite, it seems that the calcined hydrotalcite nanoparticles can cover the pores surface of the SBA-15. This assumption is supported by the fact that the average wall thickness increases from 5.3 nm (in SBA-15) to 7.0 nm. By contrast, the post-synthesized composite, in which the SBA-15 was previously calcined, HT/SBA(C), presented a BET surface area of 523 m^2^/g, such value is much lower than that of the SBA-15 and the HT/SBA(NC). The surface area diminution of the HT/SBA(C) can be attributed to the basic synthesis conditions. A basic medium can cause a partial destruction of the SBA-15 network [15]. Since the HT/SBA(NC) still presents the organic surfactant and TEOS during the coprecipitation of Mg and Al salts, the dissolution of the silanols species is not strongly affected by the basic pH, as SiO_2_ is not formed. Furthermore, during calcination, the nanocasting of Mg–Al species can occur on the mesoporous surface of the SBA-15 [9]. Indeed, the Mg–Al hydrotalcite becomes a magnesium–aluminum mixed oxide (Mg–Al–O) nanoparticles with thermal treatment and the average wall thickness increases from 5.3 to 7.0 nm [HT/SBA(NC)] or 6.2 nm [HT/SBA(C)].

In HT/SBA composites, both microporous and mesoporous coexistence follows a similar trend to the SBA-15, with a predominant mesoporous surface. The diminution of microporosity from 74 m^2^/g in SBA-15 to 37 m^2^/g (or 31 m^2^/g) may be due to the microporous blocked by Mg–Al–O particles and the calcination process. As N_2_ adsorption–desorption isotherms of HT/SBA (C) and HT/SBA (NC) composites and SBA-15 present similar profile curves (Figure 1), they can also be structurally analogous and such profiles indicate no mesoporous blocking.

The resulting textural properties are different if the composite is prepared in situ, (Mg/Al)/SBA. This composite presents a BET surface area of 806 m^2^/g with significant microporous contribution 316 m^2^/g, whereas the mesorporous surface area is 481 m^2^/g. Furthermore, the average mesoporous diameter (Ø_p_) and the average wall thickness (AWT) are smaller than those of SBA-15. Even though both materials are texturally different, as they were prepared by distinct methods, it is interesting to observe how the microporosity and mesoporosity are exalted in the in situ prepared composite. This remark is in agreement with the N_2_ adsorption–desorption behavior (Figure 1A) and the BJH pore diameter distribution (Figure 1B). Indeed, the N_2_ adsorption–desorption isotherms of the HT/SBA(C) and HT/SBA(NC) as well as their pore distribution are similar to those observed in the SBA-15. In contrast, the N_2_ adsorption–desorption isotherms and pore distribution of the (Mg/Al)/SBA composite are different with an average pore diameter of 3.7 nm, which is quite similar to the pore diameter of the calcined hydrotalcite, Table 1. Furthermore, the pore distribution is narrow (ΔØ_p_ = 2.0 nm, Figure 1B).

**Figure 2:**
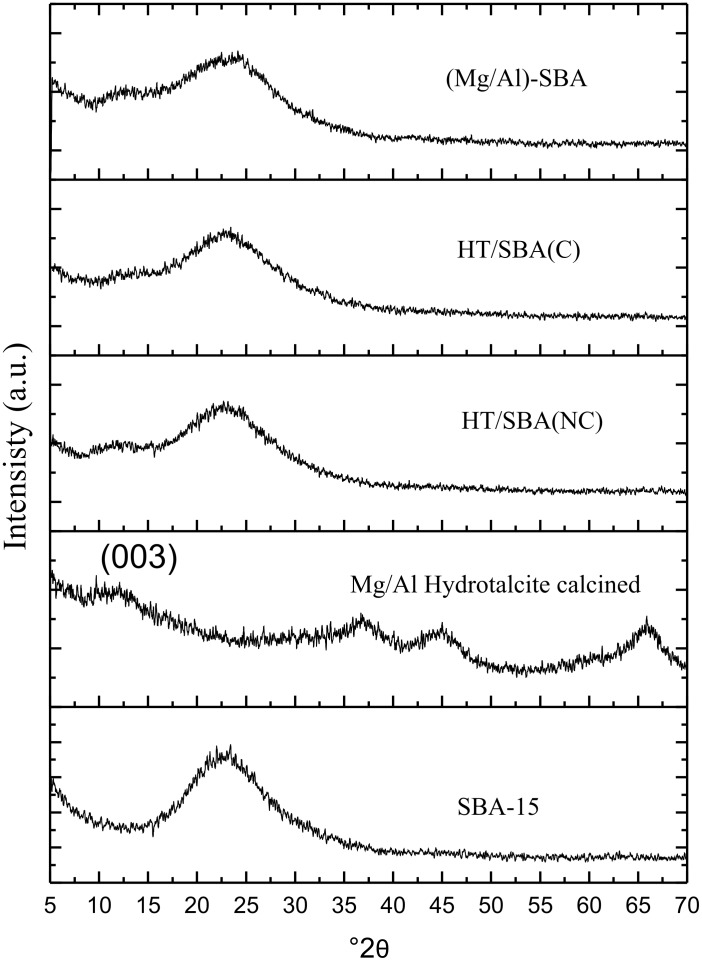
Diffractograms of X-ray of the composites, SBA-15 and HT.

Figure 2 shows that the X-ray diffractograms of all composites are similar. They present a broad and slight pick diffraction between 10° and 15° 2θ related to the (003) signal of hydrotalcites. Hence, part of the Mg–Al hydrotalcite was not fully calcined and is still preserved on the composites. Moreover, no other diffraction peaks of calcined hydrotalcie are observed by XRD, as the MgO periclase phase is only present in the calcined hydrotalcite [12], peak diffraction at 37°, 46° and 66° 2θ. Although no Mg–Al–O diffraction peaks are observed by XRD, such mixed oxides my contain particles so small that they are undetected by XRD. Indeed, the XRD patterns of composites also show a broad peak (15–30° 2θ) analogous to the amorphous SiO_2_ from SBA-15. Therefore, after calcination, the Mg–Al mixed oxides may be homogeneously dispersed without particle sintering on the surface of SBA-15. A similar result was reported by Mao et al. in a magnesium oxide-modified HZSM-5 after calcination in air at 550 °C [16].

### Morphology and Mg–Al–O dispersion

**Figure 3:**
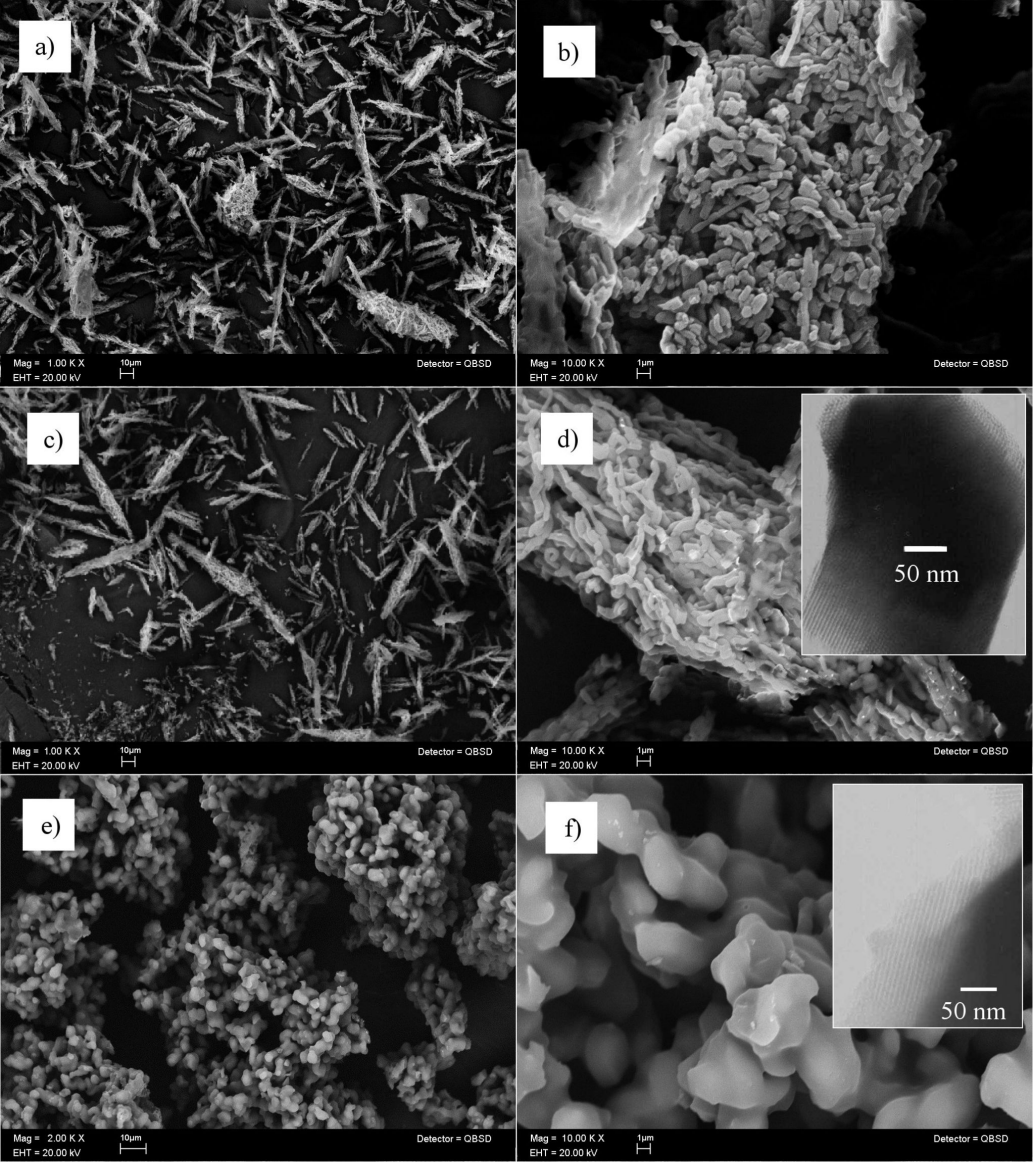
Composite SEM micrographs of a) and b) HT/SBA(C), c) and d) HT/SBA(NC), e) and f) in situ (Mg/Al)/SBA. Inset: TEM micrographs*.*

SEM micrographs of composites prepared by the post-synthesis method show a similar morphology (Figure 3a–d). Such a morphology is also observed in the pristine SBA-15 ([17] and Supporting Information File 1). The main grains present a needle-like morphology with particle sizes between 30 and 80 µm (see Figures 3a and 3c).

The needle-like particles are constituted by elongated particles of 0.5–2.0 μm, Figures 3b and 3d. Furthermore, the TEM micrograph shows pore channels, which are characteristic of the SBA-15 tubular structure (inset of Figure 3d). The morphology and distribution of Mg–Al mixed oxide nanoparticles into the SBA-15 channels cannot be observed, due the relative low scattering contrast between mixed oxides and the SBA-15 silica pore walls. Furthermore, no Mg–Al–O bulks are observed outside of the SBA-15, which is in agreement with the XRD patterns.

**Figure 4:**
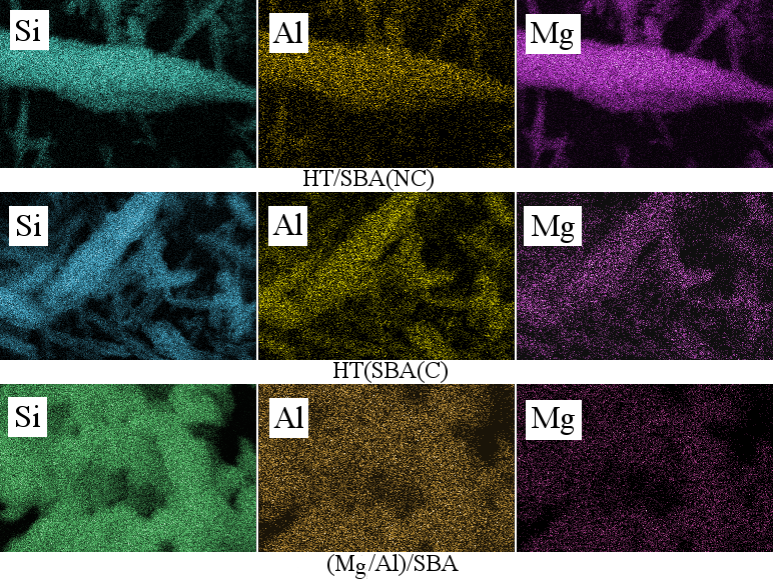
Energy dispersive X-ray (EDX) mapping analysis of composites.

The SEM micrograph of (Mg/Al)/SBA (in situ prepared sample, Figure 3e) exhibits a cauliflower-like particle arrangement, which is different from the particle arrangement observed on the pure SBA-15 and HT/SBA composites. Indeed, these “cauliflower” arrangements are constituted by smooth quasi*-*spherical particles with 2–3 μm of diameter size (Figure 3f). Despite this morphological difference, SBA-15 pore channels can be also observed by TEM microscopy, (inset of Figure 3b). Therefore, the in situ preparation method is readily achieved with a simple SBA-15 synthesis like procedure. Furthermore, the Mg, Al and Si atoms are homogeneously dispersed on the surface of all composites, as it is shown in the SEM-EDX mapping images of Figure 4.

The elemental chemical analysis evidenced that the Mg/Al bulk molar ratio of the hydrotalcite is equal to 2.03 (Table 1). This value is in agreement with its nominal composition (Mg/Al = 2). The composites prepared in post-synthesis methods showed a molar ratio of Mg/Al = 1.85 and 1.95, respectively. Such ratios are very close to the nominal composition of the calcined composites. Still, the (Mg–Al)/SBA composite presents a Mg/Al molar ratio of 1.40, which is lower than that of the nominal composition. Thus, the aluminum content is higher than the magnesium amount. These results are also in agreement with the SEM-EDX mapping images shown in Figure 4. Furthermore, these images confirm that Al and Mg oxides are homogeneously dispersed.

### Vapor sorption behavior

**Figure 5:**
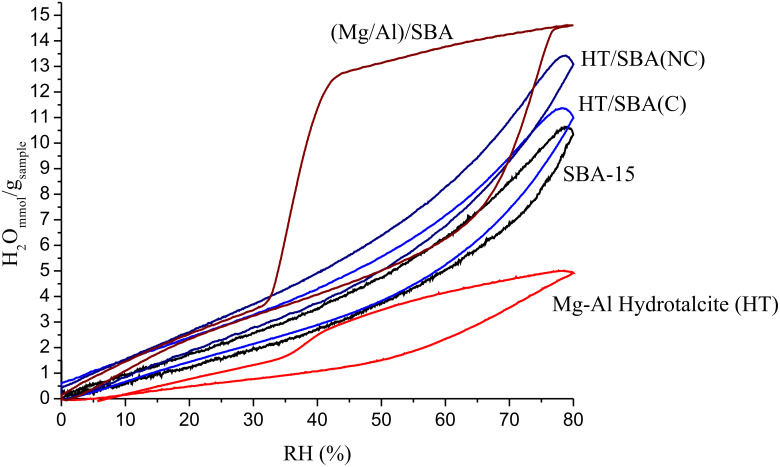
Adsorption–desorption isotherms of water vapor of the HT, SBA-15 and nanoporous composites.

The water vapor adsorption–desorption isotherms of all samples were acquired at 60 °C with a rate of 0.5%·min^−1^ of relative humidity (RH) until to achieve 80% of RH (Figure 5).

The calcined hydrotalcite (HT) adsorbs up to 5.0 mmol H_2_O/g_HT_ and the SBA-15 retains about 10 mmol of H_2_O/g_SBA-15._ Instead, the composites adsorb between 11.0 and 14.5 mmol of H_2_O/g_composite_. The composite prepared without prior calcination of the SBA-15, HT/SBA(NC), shows an increased water absorption of ca. 18% of (Δ2 mmol H_2_O/g_composite_), if compared with the water uptake of the composite prepared after the calcination of SBA-15, HT/SBA(C). The HT/SBA(NC) composite presents a BET surface area smaller than that of SBA-15 (Table 1) with 20 wt % of mixed oxides derived from hydrotalcite covering its surface. Thus, these microstructural properties and Mg–Al–O contribute to enhance the H_2_O adsorption capacity. Indeed, the SBA-15 shows a smaller amount of adsorbed water, 10.4 mmol H_2_O/g_SBA-15_, but similar textural properties, than those of the composite HT/SBA(NC) (13.5 mmol H_2_O/g_sample_). As both composites prepared by post-synthesis present a similar mesoporous distribution, they exhibit water adsorption–desporption isotherms with analogous hysteresis, as SBA-15. According to N_2_ adsorption experiments, the pore sizes of these composites are distributed around 5.7 and 4.6 nm, respectively, which produces similar water desorption rates. The increasing water amount adsorbed is favored by the Mg–Al oxide nanocasting improving the water affinity on the composite surface. This can be more visualized by the results showed for sample (Mg/Al)/SBA prepared by the in situ method, which retains 14.5 mmol of H_2_O/g_composite_, almost three times more water than the calcined hydrotalcite. This extraordinary amount of adsorbed water can also be related to the high surface area of this composite, 806 m^2^/g. Moreover, a wider hysteresis, between 80% and 40% of RH%, is observed for this sample, (Mg/Al)/SBA, Figure 5, indicating that water is slowly desorbed. Such behavior can be attributed to the porous diameter, 3.7 nm. The smaller pores size promotes the slow release of water molecules, thus, the capillarity condensation is indeed favored. Kocherbitov et al. [18] proposed a water sorption mechanism, in SBA-15 and MCM-41, which is related to the pore size. Therefore, the sorption behavior observed in the (Mg/Al)/SBA is manly, a capillary condensation in intra-wall micropores.

**Figure 6:**
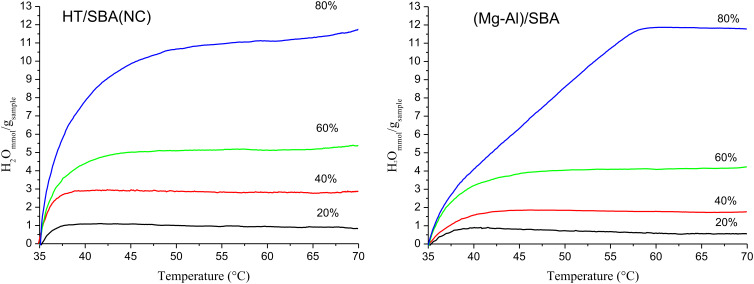
Adsorption of water vapor at different relative humidity values versus increase of temperature from 35 to 70 °C of the HT/SBA(NC) and (Mg/Al)/SBA composites.

Some experiments were carried out at different RH (20, 40, 60 or 80%) through a continuous increase of the temperature from 35 to 70 °C to determine the maximum capacity of the water vapor adsorption of the HT/SBA(NC) and (Mg/Al)/SBA composites (Figure 6).

The HT/SBA(NC) composite shows adsorption curves consistent with a Langmuir-like behavior. As expected, the amount of water vapor adsorbed increased with the RH, until equilibrium temperature is reached. Still, if the relative humidity is increased, the equilibrium is reached at high temperature. Although in the (MgAl)/SBA-15 composite the amount of water vapor adsorbed also increases with the RH, the equilibrium is reached at higher temperature than that observed for the other composite. This behavior is more pronounced at 80% RH with a linear profile from 40 °C to 55 °C (equilibrium temperature). Therefore, the smaller pore size causes slow water diffusion.

## Conclusion

Nanoporous composites were obtained from combinations of Mg–Al precursors of a hydrotalcite with SBA-15. The post-synthesis method is a simple and effective way to prepare composites that preserve the textural SBA-15 properties and also the basic properties. Indeed, Mg–Al precursors are homogenously dispersed mainly on the mesoporous SBA-15 surface. Furthermore, if the SBA-15 still contains surfactant (i.e., not calcined), the grafting through the HT coprecipitation of metal salts is as a good method to obtain a composite that is structurally stable after calcination at 550 °C and also exhibits a high water uptake. Through the in situ method, in which Mg–Al species are added during the preparation of SBA-15, a composite with cauliflower morphology and equal microporous and mesoporous contribution is produced. The in situ prepared composite was the best water adsorbant, compared to the other composites or the pristine materials. Furthermore, this sample can be prepared easily in one-step without the addition of any other chemical promoters. Finally, the highly dispersed Mg–Al–O over the SBA-15 promotes composites with a high BET specific surface area and enhanced water sorption abilities. Therefore, it can also favor, for instance, the adsorption of hydrophilic reactants in catalytic reactions, in gas storage or during the use as controlled molecular delivery materials.

## Experimental

### Synthesis of composites and precursor materials

SBA-15 was prepared as described elsewhere [19–20]. 16 g of Pluronic 123 (98%, Aldrich) were mixed with 474 mL of a 2 M HCl solution in a polyethylene recipient. The mixture was stirred at room temperature for 1 h and then at 38 °C for another hour. 34.4 mL of tetraethylortosilicate TEOS (98%, Aldrich) were slowly added to the mixture and it was stirred for 24 h at room temperature. The mixture recipient was then placed in an oven at 95 °C for 72 h. The solids were recovered by decantation, washed with distilled water, and dried at 70 °C. Half of the sample was calcined in air at 550 °C for 6 h to eliminate the organic template.

A typical Mg–Al hydrotalcite was synthesized according to the procedure reported in a previous work [21]. Two aqueous solutions, one containing Mg(NO_3_)_2_·6H_2_O and Al(NO_3_)_3_·7H_2_O (both from Aldrich, 98%), and the other NH_4_OH (2 M), were added dropwise into a flask at room temperature. The amount of each solution was calculated to obtain a Mg/Al molar ratio of 2. The addition of each solution was adjusted to pH 8. The mixture was then treated in an autoclave at 80 °C for 24 h. The solids were recovered by decantation, washed with distilled water, and dried in an oven at 70 °C.

The nanocomposites materials were prepared by combining suitable amounts of SBA-15 with a Mg–Al nitrated hydrotalcite to obtain composite materials with a 80/20 wt % ratio. The first procedure hereafter referred to as the post-synthesis method involved the dispersion of an appropriate amount of SBA-15 (80 wt %) in distilled water (25 mL/g) and the placement in a Pyrex flask. Afterwards, a 2 M solution of Mg and Al nitrated salts and a 2 M NH_4_OH solution were individually added dropwise and stirred with non-calcined SBA-15 previously dispersed in water. The amount of Mg and Al salts corresponded to a molar ratio Mg/Al of 2. The addition of each solution was adjusted to maintain a pH 8. The mixture was stirred at room temperature for 24 h, then washed with distilled water, dried overnight at 70 °C, and the template was removed by calcination at 550 °C for 6 h. This sample was labeled as HT/SBA(NC). A similar procedure was followed to prepare a second composite, but the hydrotalcite precursors were added to a dispersion of an appropriate amount of SBA-15 already calcined at 550 °C for 6 h. This sample is referred to as HT/SBA(C). A third material was prepared by an in situ method. A 2 M solution of Mg and Al nitrated salts (Mg/Al molar ratio of 2) was added dropwise into a polyethylene bottle, which contains the structuring agent of SBA-15, Pluronic 123 previously dissolved with 474 mL of a 2 M HCl solution and stirred for 24 h. After that, 34.4 mL of tetraethyl ortosilicate (TEOS) was added to the Mg–Al-Pluronic mixture and stirred for 24 h. The mixture was heated at 90 °C for 72 h, the solid was then recovered by decantation, washed with distilled water, dried at 70 °C, and calcined at 550 °C for 6 h in air. This sample is referred to as (Mg-Al)/SBA.

### Characterization techniques

X-ray diffraction (XRD) patterns were recorded with a Bruker axs D8 advance diffractometer coupled to a copper anode X-ray tube. N_2_ adsorption–desorption isotherms were measured with a Micromeritics ASAP 2020 system at −196 °C. Prior to analysis, the samples were pretreated in vacuum at 200 °C for 5 h. The total pore volume was evaluated from the desorption branch of the isotherm by using the BJH model. The *t*-plot method was useful to obtain microporous and mesoporous surfaces. Elemental analyses of Mg and Al were determined by atomic absorption (AA) with a Perkin Elmer 220 spectrometer. Scanning electron microscopy (SEM) images were recorded with a Cambridge Leica Stereoscan 440 microscope. Samples were previously coated with gold to avoid the lack of conductivity. An X-ray energy dispersive analysis (EDX) system was coupled to the SEM. Transmission electron microscopy images were recorded with a JEOL JEM-1200EX microscope operated at 120 kV. Thermogravimetric (TG) experiments were performed under air atmosphere with a heating rate of 5 °C·min^−1^ by a thermobalance provided by TA Instruments, model Q500HR. Water vapor adsorption tests were carried out on a temperature-controlled thermobalance Q5000SA from TA Instruments, equipped with a humidity-controlled chamber. The experimental variables were temperature and relative humidity (RH). Such experiments were carried out by using N_2_ from Praxair (grade 4.0) as a carrier gas and distilled water as the vapor precursor and at a total gas flow of 100 mL/min. Water vapor isotherms at 40, 50, 60, 70 or 80 °C were generated from 0 to 80% of RH. Furthermore, water vapor adsorption experiments were carried out from 35 to 70 °C at constant RH.

## Supporting Information

File 1 Additional characterization of materials by XRD, TGA and SEM-TEM analysis.
