# Supplementary material for: Nanoporous composites prepared by a combination of SBA-15 with Mg–Al mixed oxides. Water vapor sorption properties
Source: Beilstein J Nanotechnol. 2014 Aug 7;5:1226–34. doi: 10.3762/bjnano.5.136 (PMC4143113; doi:10.3762/bjnano.5.136)
Supplement: File 1 — Additional characterization of materials by XRD, TGA and SEM-TEM analysis. [file Beilstein_J_Nanotechnol-05-1226-s001.pdf]

Supporting Information

for

Nanoporous composites prepared by combination of SBA-15 with Mg–Al mixed oxides. Water vapor sorption properties

Amaury Pérez-Verdejo[1], Alvaro Sampieri[1]*, Heriberto Pfeiffer[2], Mayra Ruiz-Reyes[1], Juana-Deisy Santamaría[1] and Geolar Fetter[3]

Address: [1]Benemérita Universidad Autónoma de Puebla, Facultad de Ingeniería Química, Ciudad Universitaria, 72570, Puebla, PUE, Mexico, [2]Instituto de Investigaciones en Materiales, Universidad Nacional Autónoma de México, Circuito exterior s/n, Cd. Universitaria, Del. Coyoacán, 04510, México DF, Mexico and [3]Benemérita Universidad Autónoma de Puebla, Facultad de Ciencias Químicas, Ciudad Universitaria, 72570, Puebla, PUE, Mexico.

Email: Alvaro Sampieri - alvaro.sampieri@gmail.com

*Corresponding author

Additional characterization results

XRD diffraction

Figure S1 shows the XRD patterns at small angle 2θ of composite. If the composites are prepared by post-synthesized method, HT/SBA samples, XRD patterns show the diffraction peaks characteristic of the SBA-15, (100), (110) and (220). The (Mg/Al)/SBA exhibit a very small peaks at 1.5 º2θ. Interlayer distance (d_{100}) are presented in Table 1.

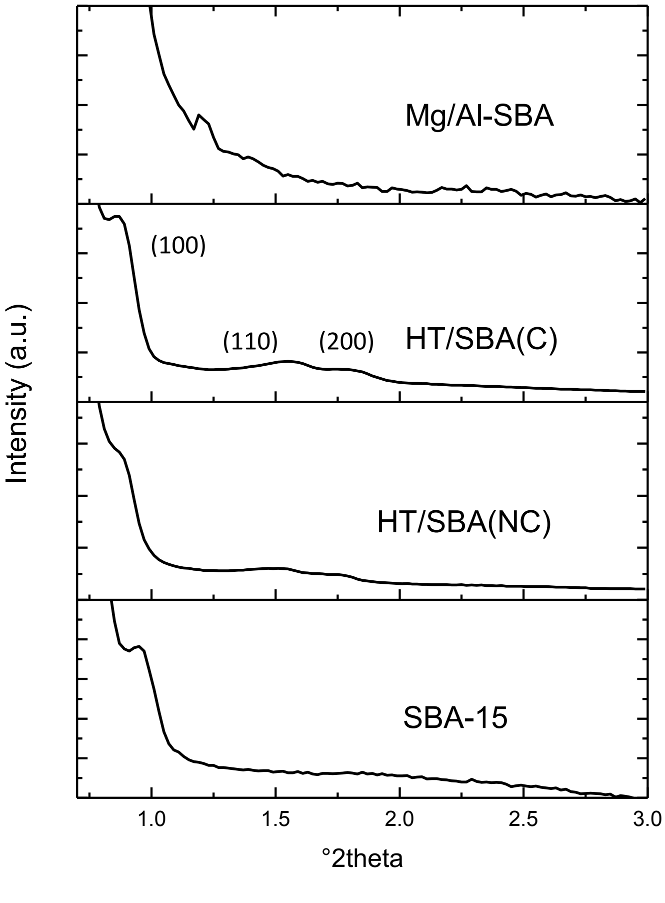

Figure S1: XRD patterns of samples at low °2θ.

Thermogravimetric analysis

Figure 3 compares the thermograms (TG) of all composites previously calcined at 550 °C. The weight loss of SBA-15 is rather insignificant (0.8 wt %), which is attributed to the water molecules desorption. Slight weight variations observed from 200 °C are due to condensation of isolated silanol groups. The *post-synthesized* composites, HT/SBA(C) and HT/SBA(NC), present ca. 4 wt % of difference. The HT/SBA(NC) composite may present more silanol groups than HT/SBA(C) [1]. Still, the Mg–Al hydrotalcite presented the most weight loss of all samples, ca.12 wt %. The in situ-synthesized composite, (Mg/Al)SBA shows a final weight loss similar to the HT/SBA(NC), ca. 8 wt %. The weight loss of composite is due to the OH decomposition from superficial $[Mg\text{-}Al(OH)_n]^{(2-n)}$ groups, which remain on the composites surface [1].

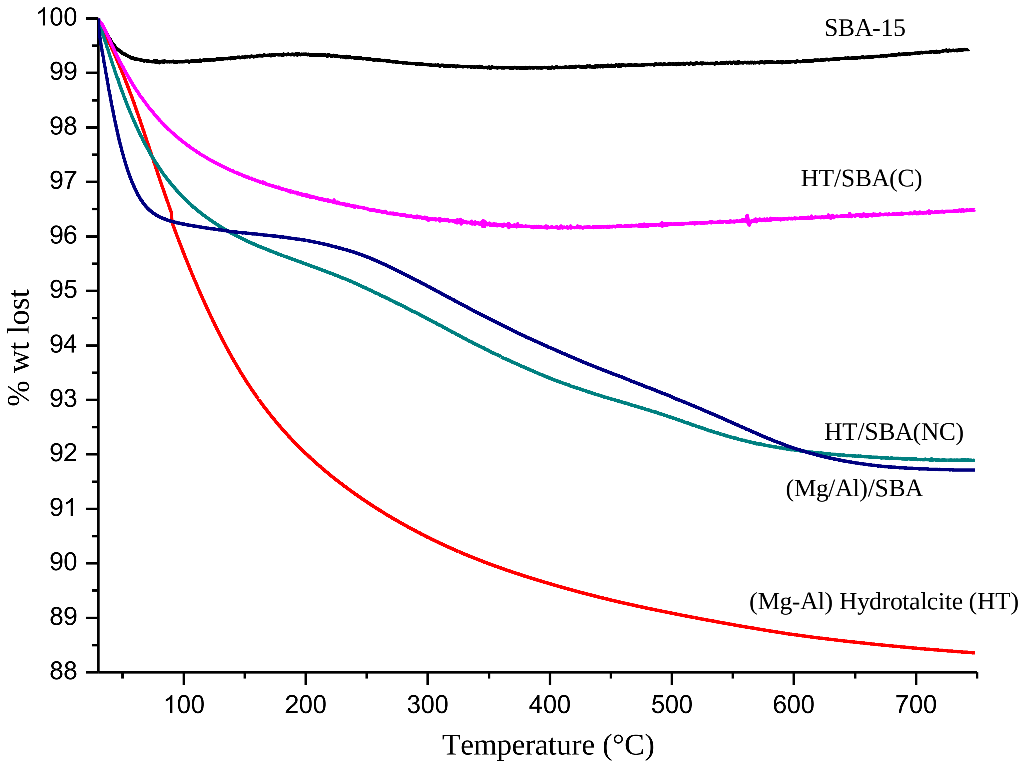

Figure S2: Thermogravimetric (TG) experiments of the HT, SBA-15 and nanoporous composites. All samples were previously calcined at 550 °C.

SEM and TEM images of SBA-15

The SEM analysis shows a SBA-15 with elongated particles having sized between 0.5 and 2 μm, Figure S3. By TEM analysis the channels characteristic of the SBA-15 are observed, Figure S4.

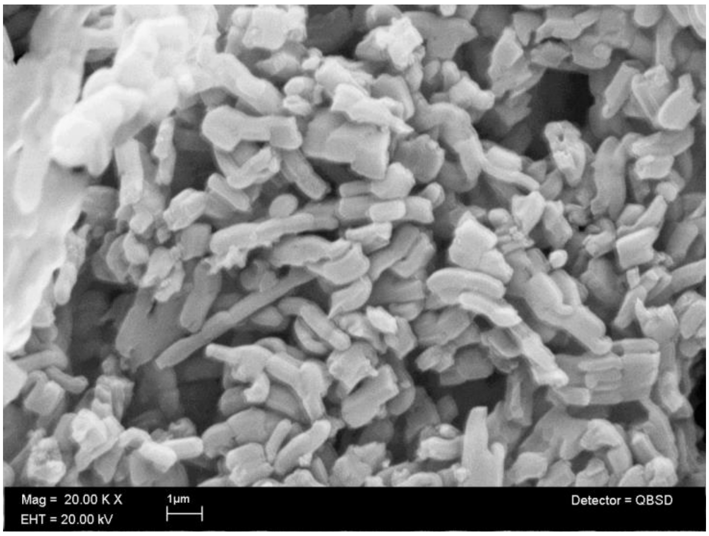

Figure S3: SEM image of SBA-15.

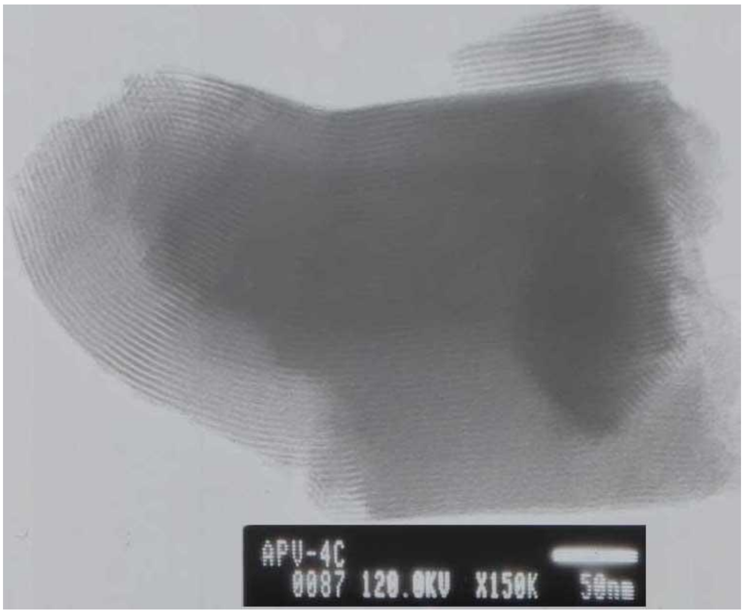

Figure S4: TEM image of SBA-15.

References

1. Ide, M.; El-Roz, M.; De Canck, E.; Vicente, A.; Planckaert, T.; Bogaerts, T.; Van Driessche, I.; Lynen, F.; Van Speybroeck, V.; Thybault-Starzyk, F.; Van Der Voort, P. *Phys. Chem. Chem. Phys.* **2013,** *15*, 642. doi:10.1039/c2cp42811c
